# Radiological–behavioral disparities in experimental osteoarthritis: Sex-specific progression and therapeutic response in a rat model

**DOI:** 10.14202/vetworld.2025.2712-2722

**Published:** 2025-09-18

**Authors:** Armando Reinaldo Marques Silva, Eduardo Rodrigues Silva, José Renzo Castro Garcês, Gabriel Moreira Pereira, Raysa Lins Caldas, Isaias Moreira de Figueiredo, Lilah Karen Ribeiro Ferreira, Davi de Sousa Pinheiro, Nathalya dos Santos Martins, Adriana Araújo Dourado, Eduardo Martins de Sousa, Maria do Socorro de Sousa Cartágenes, Rafael Cardoso Carvalho

**Affiliations:** 1 Graduate Program in Health Sciences, Center for Biological and Health Sciences, Federal University of Maranhão, São Luís, Brazil; 2 Institutional Program for Scientific Initiation Grants - PIBIC/UFMA, Center for Biological and Health Sciences, Federal University of Maranhão, São Luís, Brazil; 3 Chapadinha Science Center, Federal University of Maranhão, Chapadinha, Brazil; 4 Veterinary Imaging Diagnosis, São Luís, Brazil; 5 Program in Biosciences Applied to Health, CEUMA University, São Luís, Brazil

**Keywords:** behavioral assessment, experimental rat model, non-steroidal anti-inflammatory drugs, osteoarthritis, radiological analysis, sex differences

## Abstract

**Background and Aim:**

Osteoarthritis (OA) is a progressive degenerative joint disorder characterized by cartilage loss, subchondral bone remodeling, and chronic pain, and remains a leading cause of disability worldwide. Although radiographic imaging and behavioral testing are widely used in preclinical research, few studies have systematically examined their interdependence. This study aimed to radiologically characterize OA progression in rats induced by monosodium iodoacetate (MIA) and to correlate structural alterations with functional and nociceptive outcomes, while exploring potential sex-specific differences and therapeutic responses to non-steroidal anti-inflammatory drugs (NSAIDs).

**Materials and Methods:**

Thirty-six Wistar rats (male and female) were divided into six experimental groups: Healthy controls, OA-induced untreated, and OA-induced meloxicam-treated. Over 28 days, animals underwent serial radiological assessments and validated behavioral tests, including weight-bearing, rotarod, and Von Frey assays. Statistical analyses employed analysis of variance with *post hoc* testing, ensuring methodological rigor with blinded evaluators and sex-stratified comparisons.

**Results:**

Radiographs revealed classical OA features, joint space narrowing, subchondral bone sclerosis, and osteophyte formation, with progressive severity across timepoints. NSAID-treated males demonstrated significant improvement in motor coordination and nociceptive thresholds on days 7 and 14 (p < 0.001), whereas females exhibited only modest or delayed responses despite more severe radiological deterioration. Importantly, structural joint damage did not consistently align with behavioral impairments, underscoring a dissociation between radiographic severity and clinical-like manifestations.

**Conclusion:**

This study provides an integrated evaluation of structural and functional outcomes in experimental OA, highlighting a complex relationship between radiological changes and behavioral impairments. The findings emphasize the necessity of multimodal assessment strategies in preclinical OA models and reveal sex-specific differences in disease progression and therapeutic response. These insights are crucial for refining translational strategies, advocating for sex-conscious approaches and combined structural-functional endpoints in OA research and drug development.

## INTRODUCTION

Osteoarthritis (OA) is a progressive, chronic, and degenerative joint disease characterized by cartilage loss and subchondral bone remodeling of multifactorial etiology, characterized by arthralgia, stiffness, limited joint function, and progressive cartilage loss with inadequate repair [1]. It is one of the most prevalent musculoskeletal disorders worldwide, representing the leading cause of disability in elderly individuals and the fourth leading cause in women [2]. OA-related medical expenditures constitute approximately 1%–2.5% of the gross domestic product (GDP) annually in several countries, with hip and knee joint conditions contributing the most to these expenses [3, 4].

According to the Brazilian Society of Rheumatology [5], OA primarily affects the hip joint and is rare in individuals aged 40 years, becoming increasingly prevalent after age 60 years. Approximately 85% of individuals present radiological or clinical evidence of OA by the age of 75; however, only 30%–50% of those with radiographic changes report chronic pain. Given these factors, OA poses a significant challenge for public health policies, representing both a growing concern in medicine and a critical research priority [6]. Experimental animal models are essential in OA research, enabling controlled investigation of disease mechanisms and progression [7, 8]. These models are widely used across various species, particularly rats and mice, and are broadly accepted by the global scientific community [9]. Among the available models, the monosodium iodoacetate (MIA)-induced model in rats has gained prominence due to its rapid induction of joint degeneration, cost-effectiveness, and reproducibility. MIA causes chondrocyte death by inhibiting glyceraldehyde-3-phosphate dehydrogenase, consistent cartilage degradation, and subchondral bone alterations [8]. Compared with surgical models [10], such as anterior cruciate ligament transection, meniscectomy, or destabilization of the medial meniscus, the MIA model is less invasive, more standardized, and mimics the inflammatory and nociceptive components of OA with high fidelity. In addition, it allows for temporal control of OA severity and has been extensively validated for assessing both structural changes and pain-related behaviors [11–13]. The MIA rat model was selected due to its reproducibility, relevance to human OA pathology, and well-documented pain and inflammatory responses.

Previous studies by Sophocleous and Huesa [10], Rotpenpian *et al*. [14], Wang *et al*. [15] and Artuzi *et al*. [16] have demonstrated the efficacy of laboratory animals in OA research, highlighting the applicability of experimental models in evaluating functional, radiological, and microscopic joint alterations. While a previous study by Segal and Anderson [17] predominantly utilized radiography for diagnostic purposes, our study innovatively correlates radiographic features directly with behavioral analysis tools for OA diagnosis, with radiography being the most widely used due to its accessibility and cost-effectiveness.

Radiological criteria for OA diagnosis and disease progression assessment in experimental models are primarily based on those established for humans by Kellgren and Lawrence [18]. However, their applicability in animal models remains questionable. With extensive experience in this experimental model, our research group has observed considerable variability in the correlation between radiological findings and pain assessments in animals. These discrepancies likely arise from significant anatomical and physiological differences between species, emphasizing the need for a more precise and species-specific approach in evaluating experimental OA. Despite the widespread use of behavioral and radiological assessments in OA models, few studies have systematically examined the degree of correlation between these two domains. Most preclinical studies focus on structural deterioration or nociceptive behavior, but rarely on their interdependence. This creates a significant gap in understanding how radiographic changes reflect functional impairment *in vivo*.

This study explicitly addresses this gap by integrating longitudinal radiological assessments with validated behavioral tests in a rat model of controlled MIA. Furthermore, by including both sexes and analyzing potential sex-dependent differences, this study contributes novel insights into the multifactorial progression of OA and its diagnostic interpretation, ultimately informing translational strategies for more effective therapeutic evaluation. The inclusion of both male and female subjects is particularly relevant given the growing recognition of sexual dimorphism in the incidence, progression, and perception of OA. Epidemiological data indicate that women, especially those who are postmenopausal, are disproportionately affected by OA, often presenting with more severe symptoms and functional impairment [3, 19]. These differences may stem from hormonal, genetic, and biomechanical factors and are rarely accounted for in preclinical studies by Serra and Soler [7] and Stürmer *et al*. [20]. This study seeks to elucidate whether sex influences OA manifestation and therapeutic response by evaluating radiological and behavioral parameters in both sexes, thereby aligning preclinical research with precision medicine principles.

## MATERIALS AND METHODS

### Ethical approval

All experimental procedures were conducted in strict accordance with the ethical standards established by CONCEA and SBCAL. The study protocol was reviewed and approved by the Ethics Committee on Animal Use of the Federal University of Maranhão (Protocol No. 23115.002825/2023-71). All animals were maintained under controlled environmental conditions (24 ± 2°C, 55 ± 5% humidity, and a 12 h light/dark cycle) with free access to food and water throughout the 28-day experimental period.

### Study period and location

The study was conducted from July to August 2024 at the Animal House and Experimental Center (BCEA) and the Laboratory of Experimental Pain Studies (LEED), both at the Federal University of Maranhão (UFMA), São Luís, Brazil.

### Animal experimentation

#### Anesthetic protocol and induction of OA

Healthy Wistar rats (*Rattus norvegicus*), aged 90 days and weighing 250 g–350 g, were obtained from the Bioterium and Animal Experimentation Center of the Federal University of Maranhão (BCEA/UFMA). Animals were housed under standardized conditions (24°C ± 2°C; 55% ± 5% humidity; 12-h light/dark cycle) with ad libitum access to standard chow and water. A 7-day acclimatization period in the BCEA/UFMA preceded experimental procedures.

OA was induced by an intra-articular injection of MIA, 2 mg/kg, which was selected for its ability to consistently reproduce cartilage degeneration with minimal systemic effects. The injection was performed under knee flexion, using the lateral patellar ligament as a landmark to ensure precision [11–13]. General anesthesia was induced intraperitoneally with ketamine hydrochloride (50 mg/kg), midazolam hydrochloride (1 mg/kg), and tramadol hydrochloride (5 mg/kg), and maintained with isoflurane (2.5%) delivered through a mask.

#### Experimental protocol

A controlled preclinical trial design was adopted. Thirty-six rats were randomly allocated into six groups (n = 6/group):

- Healthy male group (no OA induction)
- Healthy female group (no OA induction)
- OA-induced male group (untreated)
- OA-induced male group treated with meloxicam (1 mg/kg/day, oral gavage for 28 days, starting 1 day post-induction)
- OA-induced female group (untreated)
- OA-induced female group treated with meloxicam (same regimen as males).

Sample size was determined using G*Power software based on a one-way analysis of variance (ANOVA) design, with parameters set at α = 0.05, power (1−β) = 80%, and effect size (Cohen’s f) = 0.4, which is representative of a large effect. This calculation indicated the need for six animals per group, balancing statistical sensitivity with ethical principles of animal use (3Rs).

The meloxicam dosage was selected based on pharmacological evidence and preclinical OA studies in rodents, demonstrating systemic anti-inflammatory and analgesic efficacy at this concentration while minimizing adverse effects [6, 12, 16, 21, 22]. Oral gavage was chosen for its consistency with human clinical practice and reduced stress during repeated administration.

### Biomechanical and behavioral tests

Behavioral assessments were conducted at baseline (day 0) and on days 7, 14, 21, and 28. All tests were performed in a temperature-controlled room (22°C–24°C), at the same time of day, by three blinded evaluators to minimize bias.

#### Weight-bearing test (WBT)

The static WBT was used to assess hind limb load distribution as an indirect measure of joint discomfort and primary hyperalgesia. The apparatus (Insight®, Brazil) included two independent force transducers, which recorded hind limb weight (g) over a 5-s period while the animal remained in an acrylic chamber. Each animal underwent three valid trials (≥1 min apart), and the mean value was used for analysis [11, 12].

Weight distribution (%) was calculated as:

Weight distribution (%) = PPA/(PPA + PPC) × 100

Where, PPA = weight supported by the affected limb and PPC = weight supported by the contralateral limb.

#### Von Frey test

Mechanical nociceptive thresholds were assessed using calibrated Von Frey filaments (4 g, 10 g, 300 g). Rats were acclimated for 30 min in acrylic enclosures placed on a wire mesh platform before testing. The 10 g filament was applied first; subsequent filament strength was adjusted based on responses:

- Positive response (paw withdrawal, licking, or biting): lower-force filament applied
- Negative response: higher-force filament applied

Each trial was separated by ≥30 s. Testing continued until three consecutive consistent responses were obtained. Lack of response was recorded as negative [11]. Data were averaged across three blinded evaluators, and paw withdrawal nociceptive threshold (PWNT) was calculated as:

PWNT (%) = LNRPA/(LNRPA + LNRPC) x 100

Where, PWNT = paw withdrawal nociceptive threshold; LNRPA = threshold for the affected paw; LNRPC = threshold for the contralateral paw.

#### Rotarod test

Motor coordination and limb use were assessed using a Rotarod device (30 cm diameter, stainless steel mesh surface) rotating at 4–40 rpm for 300 s. Animals underwent two training sessions before baseline testing. During the test, latency to fall and limb-use impairment were scored on a 5-point scale:

- 5 = normal use; 4 = mild limping; 3 = severe limping; 2 = intermittent disuse; 1 = complete disuse

Each rat was tested 3 times per session (≥15 min apart), and mean scores were calculated by three blinded evaluators [12].

### Radiographic evaluation

Radiographic imaging of rat knee joints was performed using the Micro Imagem Diox-602- X-ray System (DigiMed Co., Ltd., South Korea) (60 kV, 4 mA) with a digital sensor. Animals were anesthetized (isoflurane 2.5%) and positioned in dorsal and left lateral recumbency. Images were acquired in craniocaudal and laterolateral projections with knees extended and flexed. Radiographs were obtained on days 0, 4, 7, 14, 21, and 28. All images were analyzed by a veterinary imaging specialist blinded to treatment and sex.

### Statistical analysis

Data were analyzed using GraphPad Prism 10.0 (GraphPad Software, San Diego, CA, USA). One-way ANOVA followed by Tukey’s *post hoc* test was applied for comparisons among multiple groups. Two-way ANOVA was used for variables involving two independent factors (treatment and sex). Data normality was assessed using the Shapiro–Wilk test. A significance threshold of p < 0.05 was adopted for all analyses.

## RESULTS

### Behavioral assessments

#### Rotarod test – Motor performance

**Figure 1:**
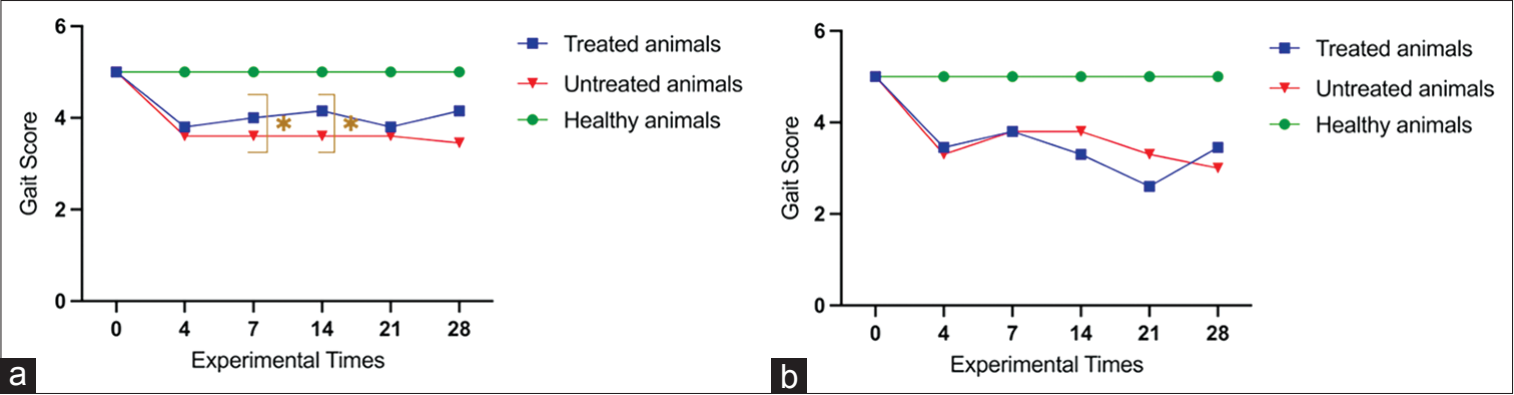
Behavioral assessment using the rotarod test in rats subjected to the monosodium iodoacetate-induced experimental osteoarthritis protocol. Analysis was conducted at experimental time points 0 (pre-induction), 4, 7, 14, 21, and 28 days post-induction and treatment. (a) Analysis of male and (b) female animals. Data are presented as mean values for each group. p < 0.001 – Comparison between treated and untreated animals subjected to the experimental protocol.

Motor coordination was evaluated using the rotarod test at multiple time points during the experimental protocol (Figure 1). On day 14, healthy male and female rats maintained the highest performance scores (mean = 5.0; SD = 0.0), indicating preserved motor function and absence of discomfort. In contrast, OA-induced untreated males exhibited marked motor impairment (mean = 2.1; standard deviation [SD] = 0.4; 95% confidence interval [CI]: 1.8–2.4), whereas NSAID-treated males showed partial functional recovery (mean = 3.8; SD = 0.5; 95% CI: 3.4–4.2), consistent with an analgesic response. Statistical analysis confirmed a significant difference between treated and untreated males on days 7 and 14 (p < 0.001).

In females, untreated animals demonstrated reduced motor function (mean = 2.2; SD = 0.5; 95% CI: 1.8–2.6). Treated females exhibited only a modest improvement (mean = 2.6; SD = 0.6; 95% CI: 2.1–3.1), which did not reach statistical significance at early time points. Graphical trends indicated possible improvement from day 21 onward, though this was insufficient to achieve significance.

#### Statistical validation of rotarod data

Normality testing with the Shapiro–Wilk test showed that treated male rats conformed to a normal distribution (W = 0.8456; p = 0.1449), allowing parametric analysis. Conversely, untreated males deviated from normality (W = 0.7914; p = 0.0491), necessitating non-parametric methods. A similar pattern was seen at earlier time points (treated: W = 0.8220, p = 0.0918; untreated: W = 0.6717, p = 0.0031). Data from healthy controls were incomplete or statistically invalid, limiting their inclusion in comparative analyses. These findings emphasize the need for parametric approaches in treated groups and non-parametric approaches in untreated groups. Overall, the rotarod results demonstrated a therapeutic benefit of NSAIDs in males but limited efficacy in females, suggesting sex-dependent functional outcomes.

#### Von Frey test – Nociceptive thresholds

**Figure 2:**
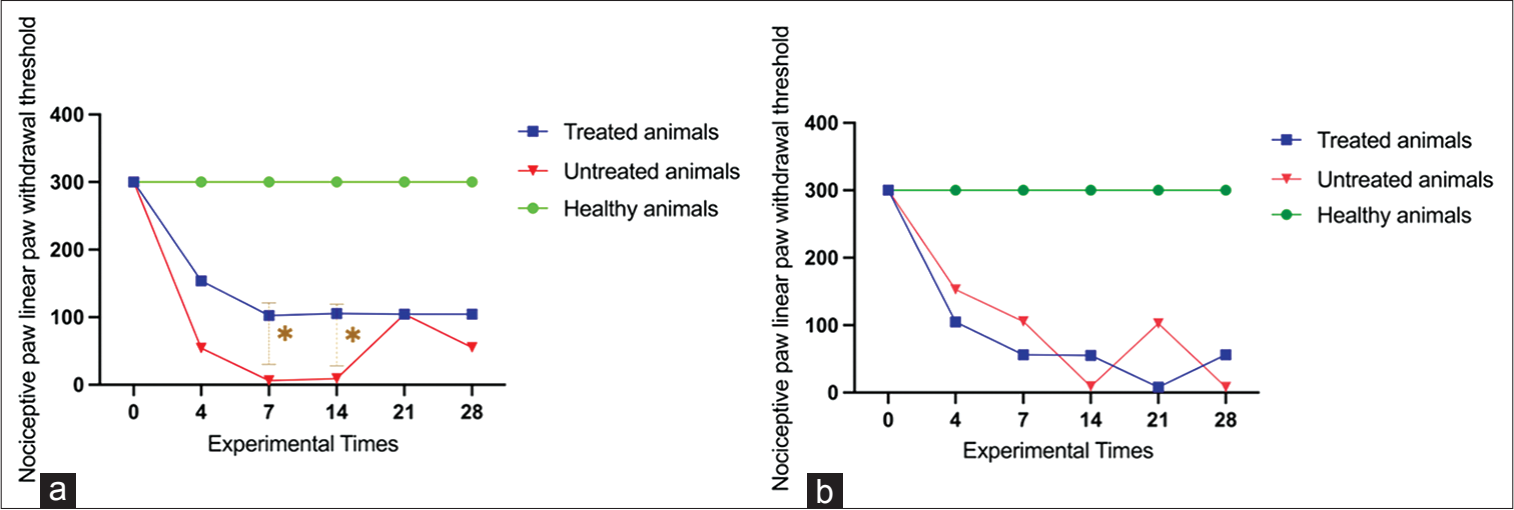
Behavioral assessment using the Von Frey test in rats subjected to the monosodium iodoacetate-induced experimental osteoarthritis protocol The analysis was conducted at experimental time points 0 (pre-induction), 4, 7, 14, 21, and 28 days post-induction and treatment. (a) Analysis of male and (b) female animals. Data are presented as mean values for each group. p < 0.001 – Comparison between treated and untreated animals subjected to the experimental protocol.

Mechanical nociceptive thresholds were evaluated using the Von Frey test (Figure 2). At baseline, both healthy males and females exhibited maximal scores (mean = 100%; SD = 0.0), confirming absence of mechanical hypersensitivity. By day 14, untreated OA-induced males showed a significant reduction in nociceptive threshold (mean = 42.5%; SD = 12.0; 95% CI: 34.4–50.6), consistent with hypersensitivity. NSAID-treated males improved markedly (mean = 76.0%; SD = 10.5; 95% CI: 69.5–82.5; p < 0.05), confirming analgesic efficacy.

In females, untreated OA animals demonstrated reduced thresholds (mean = 45.2%; SD = 11.5; 95% CI: 36.7–53.7), while treated females showed only a slight increase (mean = 48.1%; SD = 12.3; 95% CI: 39.2–57.0), with no significant difference compared to untreated counterparts.

#### Statistical validation of Von Frey data

The Shapiro–Wilk test confirmed normal data distribution in both treated and untreated males (W = 0.9169, p = 0.4833; W = 0.9252, p = 0.5436) and females (treated: W = 0.9830, p = 0.9655; untreated: W = 0.9026, p = 0.3896). Thus, parametric tests were applied. Data from healthy controls were excluded due to inconsistencies but provided reference values. Collectively, these findings indicate that NSAID treatment restored nociceptive thresholds in males but failed to improve pain sensitivity in females, reinforcing sex-related differences.

### Radiological findings

Radiographic evaluations were conducted on day 28 using craniocaudal and laterolateral projections. Across all OA-induced groups, the main findings included joint space narrowing, subchondral bone sclerosis, and marginal osteophyte formation – hallmarks of OA.

Quantitative grading with the Kellgren–Lawrence (KL) system showed progressive deterioration. In males, untreated rats had a mean KL score of 3.5 (SD = 0.3; 95% CI: 3.3–3.7), whereas treated males showed partial preservation of joint structure (mean = 2.7; SD = 0.4; 95% CI: 2.4–3.0). In females, untreated animals displayed the highest severity scores (mean = 3.9; SD = 0.2; 95% CI: 3.7–4.1), while treated females showed moderate improvement (mean = 3.3; SD = 0.3; 95% CI: 3.0–3.6), although degenerative signs persisted.

These findings suggest possible sex-related differences in disease progression and therapeutic responsiveness. Despite structural preservation in some treated animals, osteoarthritic features remained evident, demonstrating a disconnect between radiographic changes and behavioral outcomes, particularly in females.

#### Progression of radiographic features

**Figure 3:**
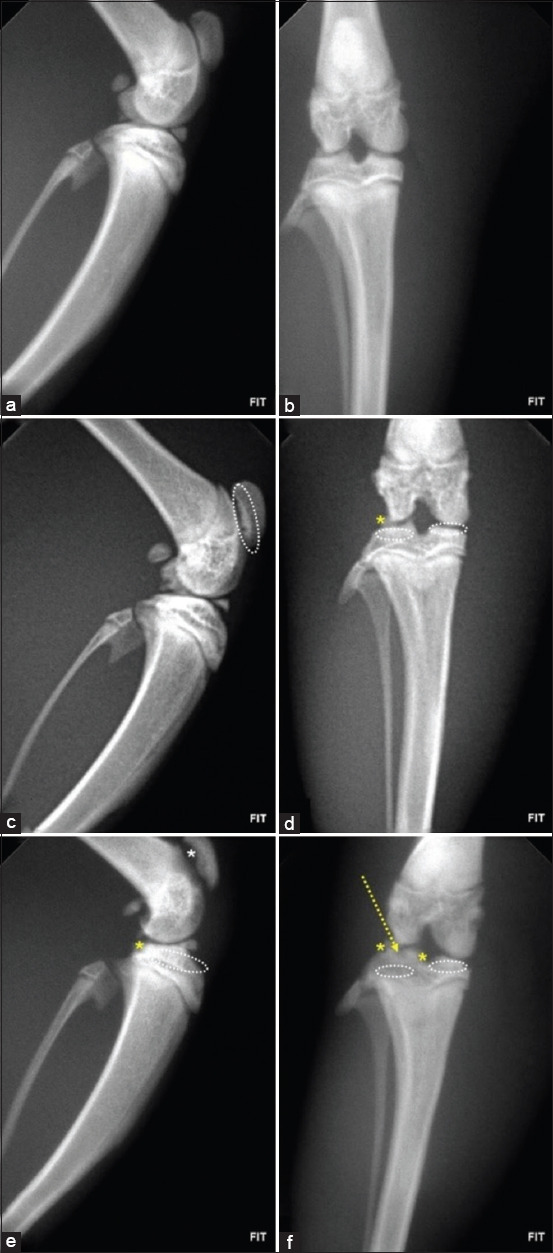
Radiographic evaluation of the knee joint in female rats in groups II, V, and VI. (a and b) Healthy animals with no radiological alterations. (c and d) Animals treated. Note the presence of marginal osteophytes on the lateral surface of the tibia (yellow asterisk) and subchondral bone sclerosis (white circles), along with narrowing of the joint space. (e and f) Untreated animals. Observe the marginal osteophytes on the lateral and medial surfaces of the tibial lateral epicondyle (yellow asterisks), leading to a loss of its normal anatomical structure (yellow arrow) and narrowing of the joint space. The articular surface alteration of the patella (white asterisk) indicates greater disease severity. Left: Laterolateral projection. Right: Craniocaudal projection.

**Figure 4:**
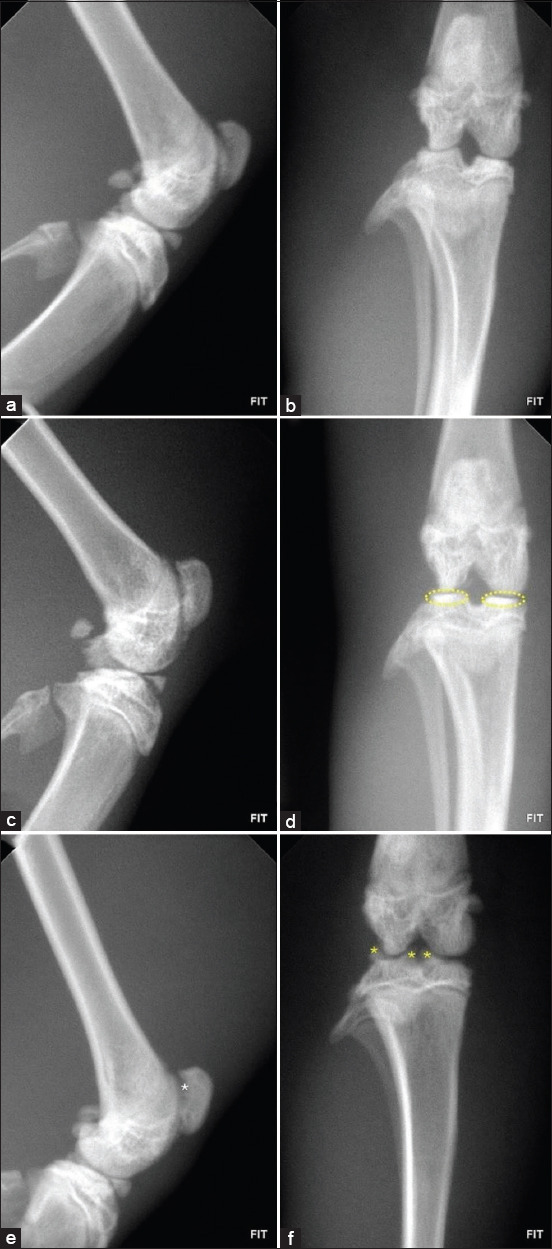
Radiographic evaluation of the knee joint in male rats: Groups I, III, and IV. (a and b) Healthy animals, showing no radiological alterations. (c and d) Animals treated. Note the presence of subchondral bone sclerosis (yellow circles) and narrowing of the joint space. (e and f) Untreated animals. Observe the marginal osteophytes on the lateral and medial surfaces of the lateral epicondyle and on the medial surface of the medial epicondyle of the tibia (yellow asterisks). Left: Laterolateral projection. Right: Craniocaudal projection.

- Healthy animals (Figures 3a and b – female group; Figures 4a and b – male group).
- Early stage (Figures 3c and d, 4c and d): Subchondral bone sclerosis appeared as radiopaque areas, indicating early bone densification.
- Moderate to advanced stage (Figures 3e and f, 4e and f): Pronounced sclerosis, joint space narrowing, and marginal osteophytes developed, reflecting cartilage loss and advanced degeneration.

These results confirm OA progression as a nonlinear process, with sclerosis and osteophytes becoming more prominent in later stages.

### Statistical analysis

All analyses were conducted using GraphPad Prism 10.0 (GraphPad Software, San Diego, CA, USA). One-way ANOVA with Tukey’s *post hoc* test was used for group comparisons, while two-way ANOVA assessed factors involving treatment and sex. Significance was set at p < 0.05.

Normality was verified using the Shapiro–Wilk test. Parametric analyses were applied to datasets that met the normality assumption (e.g., Von Frey in both sexes, rotarod in treated males). Non-parametric alternatives (Kruskal–Wallis with Dunn’s *post hoc* tests) were used for datasets that violated normality assumptions (e.g., rotarod in untreated groups). Descriptive statistics (mean, SD, 95% CI) supported transparency and interpretation of results.

## DISCUSSION

The findings of this experimental OA study provide a comprehensive analysis of structural and functional alterations associated with disease progression [7, 23]. The integration of radiological parameters and behavioral assessments provides a comprehensive perspective, aligning with existing literature while highlighting functional impairments at various stages of the experimental model.

### Behavioral assessments

#### Rotarod test

Behavioral tests, such as Rotarod and Von Frey are widely used to assess motor function and nociception in OA models. In the rotarod test, no significant differences were observed between treated and untreated groups (p = 0.6515), suggesting that NSAID treatment did not reverse functional impairments in advanced OA stages. This aligns with Bliddal [24], who emphasized the difficulty of restoring motor function without targeted interventions. However, significant differences were detected when comparing treated and untreated groups with healthy controls (p = 0.0058 and p = 0.0010, respectively), reinforcing that OA markedly impairs mobility.

These findings are consistent with Hunter and Bierma-Zeinstra [3], who reported a direct relationship between joint degradation and functional impairment. Mobility loss observed in OA models stems primarily from cartilage damage and joint inflammation, hallmarks of advanced disease [7, 25].

#### Von Frey test

The Von Frey test was used to evaluate pain sensitivity. Normality testing confirmed that both treated and untreated groups, in both sexes, followed a normal distribution, allowing parametric analysis. No significant differences in nociceptive thresholds were observed between NSAID-treated and untreated animals. This may reflect variability in OA-associated pain responses, as also noted by Alves-Simões [9], who highlighted challenges in measuring nociception, particularly in early or moderate disease stages.

Nevertheless, comparisons with healthy controls showed marked differences in sensitivity, supporting the model’s validity. Pritzker *et al*. [26] emphasized that pain sensitivity and functional loss are critical markers for validating OA models and therapeutic interventions.

### Discrepancy between structure and function

Our results revealed a consistent mismatch between structural joint damage and functional or nociceptive impairment, particularly in females. Biological and methodological factors may explain this divergence. Biologically, pain perception depends not only on cartilage loss but also on synovial inflammation, bone remodeling, and sensitization mechanisms [17, 27]. Animals may therefore present severe joint degeneration without proportional behavioral impairment. In addition, estrogen and other sex hormones may modulate pain and joint remodeling, contributing to sex-related variability [3].

Methodologically, validated behavioral tests such as Rotarod and Von Frey may show ceiling or floor effects depending on disease severity. Moreover, radiographic grading systems like KL capture late-stage alterations but may not reflect early biochemical or inflammatory changes. This temporal mismatch could partly explain the weak correlations observed. Collectively, these findings underscore the importance of developing multimodal and time-sensitive assessment strategies in OA research, which should incorporate imaging, behavioral, and molecular endpoints.

### Radiological findings

Radiological data demonstrated progressive OA-related changes. Early subchondral sclerosis (day 4) indicated an adaptive bone response to cartilage degeneration, consistent with the findings of Abramoff and Caldera [25]. Joint space narrowing appeared at later stages (days 14, 21, 28), confirming progressive cartilage loss and its role as a hallmark of OA severity [18, 28].

Osteophytes, observed at the femoral and tibial condyles, became more pronounced in advanced stages, reflecting compensatory remodeling. While osteophytes help stabilize joints, they also restrict movement and exacerbate discomfort, as described by Pritzker *et al*. [26] and Hunter and Bierma-Zeinstra [3].

Bone cysts, detected in some animals at day 21, indicated necrosis or remodeling linked to mechanical overload, consistent with Liao *et al*. [22], who associated cysts with instability and structural compromise in OA.

### Integration of structural and functional data

Integrating radiological and behavioral results revealed critical insights. Joint space narrowing strongly correlated with impaired rotarod performance, consistent with studies by Bliddal [24] and Alves-Simões [9], who linked cartilage loss to stiffness and motor deficits. Subchondral sclerosis, though an early marker, may contribute to pain sensitivity despite no clear differences between treatment groups. Osteophytes, meanwhile, were associated with advanced-stage motor impairment and pain, reinforcing their role in worsening OA symptoms [26, 29].

### Sex-dependent differences

Clear sex-based differences emerged. Male rats treated with NSAIDs demonstrated improved pain thresholds and motor function, whereas females showed limited or delayed benefits despite more severe radiological damage. This dissociation suggests underlying sex-specific mechanisms in nociception and therapeutic response.

Estrogen likely plays a role, given its effects on inflammation, bone remodeling, and pain perception. Estrogen deficiency in postmenopausal women is linked to OA severity, and similar mechanisms may exist in rodent models [3]. Furthermore, evidence suggests that females may exhibit enhanced activity of pain-related pathways, such as transient receptor potential vanilloid 1, Toll-like receptor 4, and nuclear factor-kappa B, which amplifies nociceptive responses and alters drug metabolism [27].

## CONCLUSION

This experimental study provided a comprehensive evaluation of OA progression using the MIA-induced rat model by integrating radiological assessments with validated behavioral tests. The findings demonstrated that while radiological changes, including subchondral sclerosis, joint space narrowing, osteophyte formation, and bone cysts, clearly reflected structural deterioration, behavioral outcomes such as motor impairment (rotarod) and nociception (Von Frey thresholds) showed variable correspondence. Notably, NSAID treatment did not significantly reverse functional impairment or pain sensitivity, particularly in advanced stages of OA, reinforcing the limitations of symptomatic therapy in chronic disease. Sex-based differences were evident, with male rats exhibiting more favorable responses to pharmacological treatment compared with females, despite females presenting more severe structural alterations.

The practical applicability of these findings lies in the validation of combined radiological and behavioral endpoints as reliable indicators of OA progression and therapeutic response. This integrated approach more closely mirrors clinical scenarios where structural and symptomatic outcomes must be considered together. Moreover, the observed sex-related variability highlights the importance of including both sexes in preclinical models to ensure translational relevance and to guide precision medicine strategies.

The strengths of this study include its longitudinal design, the incorporation of both sexes, and the simultaneous evaluation of structural and functional parameters, which provide a holistic framework for OA research. However, some limitations must be acknowledged. Radiographic analyses such as the KL scale capture only late-stage changes and may underestimate early synovial or biochemical alterations. Behavioral tests, while validated, are subject to ceiling/floor effects and individual variability, which may obscure subtle differences in nociception or motor function.

For future research, multimodal assessment strategies that combine radiology with molecular biomarkers, advanced imaging techniques (e.g., micro-CT or MRI), and broader behavioral assays should be pursued. Studies focusing on the mechanistic basis of sex-dependent differences, particularly the role of estrogen, inflammatory mediators, and pain-related signaling pathways, are needed to advance sex-specific therapeutic interventions.

This study reinforces the value of the MIA-induced rat model as a reproducible and translationally relevant tool for OA research. The integration of radiological and behavioral assessments provides a more complete picture of disease progression, underscores the limited efficacy of current NSAID-based therapies in advanced stages, and highlights the critical role of sex as a biological variable. Collectively, these findings support the adoption of multimodal and sex-inclusive approaches in both preclinical and clinical OA research, ultimately guiding the development of more effective and personalized therapeutic strategies.

## AUTHORS’ CONTRIBUTIONS

RCC, MSSC, and EMS: Conceptualized, designed, and performed the experiments, collected the data, and drafted and revised the manuscript. ARMS, ERS, JRCG, GMP, RLC, IMF, LKRF, DSP, NSM, and AAD: Performed the experiments, collected data, and drafted the manuscript. All authors have read and approved the final manuscript.
